# The Thoracic Shape of Hominoids

**DOI:** 10.1155/2014/324850

**Published:** 2014-04-09

**Authors:** Lap Ki Chan

**Affiliations:** ^1^Department of Biological Anthropology and Anatomy, Duke University Medical Center, Durham, NC 27708, USA; ^2^Institute of Medical and Health Sciences Education, and Department of Anatomy, The University of Hong Kong, Hong Kong

## Abstract

In hominoids, the broad thorax has been assumed to contribute to their dorsal scapular position. However, the dorsoventral diameter of their cranial thorax was found in one study to be longer in hominoids. There are insufficient data on thoracic shape to explain the relationship between broad thorax and dorsal scapular position. The current study presents data on multilevel cross-sectional shape and volume distribution in a range of primates. Biplanar radiographs of intact fluid-preserved cadavers were taken to measure the cross-sectional shape of ten equally spaced levels through the sternum (called decisternal levels) and the relative volume of the nine intervening thoracic segments. It was found that the cranial thorax of hominoids is larger and broader (except in the first two decisternal levels) than that of other primates. The cranial thorax of hominoids has a longer dorsoventral diameter because the increase in dorsoventral diameter caused by the increase in the volume of the cranial thorax overcompensates for the decrease caused by the broadening of the cranial thorax. The larger and broader cranial thorax in hominoids can be explained as a locomotor adaptation for scapular gliding and as a respiratory adaptation for reducing the effects of orthograde posture on ventilation-perfusion inequality.

## 1. Introduction

A dorsal scapular position is one of the postcranial characteristics of hominoids [1–6]. However, the adaptive significance of such a dorsal scapular position is poorly understood. Moreover, it is not entirely clear which features, and especially which thoracic features, have contributed to the formation of the dorsal scapular position.

In the brachiation theory of hominoid evolution, the dorsal scapular position of hominoids is postulated to enhance shoulder mobility, which in turn is postulated to facilitate brachiation [7–10]. The specialized glenohumeral joint of hominoids is also assumed to contribute to this increased shoulder mobility [3, 11–13]. However, in the slow climbing theory of hominoid evolution, this assumed increase in shoulder mobility has been proposed as an adaptation for slow climbing instead of brachiation, because a similar suite of postcranial features is also found in the slow-climbing lorines and* Alouatta* [1].

However, Chan [14] showed that the hominoid glenohumeral joint is actually* less* mobile than those of nonhominoid primates. Instead, the hominoid glenohumeral joint is characterized by a smooth excursion, even when the humerus is fully abducted in the plane of the scapula (in many other primates, the superior lip of the glenoid fossa wedges into the bicipital groove when the humerus moves into that position). Chan [15] further showed that nonhylobatid hominoids do not have higher shoulder mobility in the craniodorsal directions than arboreal quadrupedal monkeys and that the lorines do not have greater craniodorsal mobility than arboreal quadrupedal prosimians. These findings indicate that the hominoids' dorsal scapula has not resulted in a higher shoulder mobility. Therefore, they cast doubt on the explanation of the dorsal scapular position in hominoids as a mobility-enhancing feature for brachiation or slow climbing.

As for the structures contributing to the formation of the dorsal scapular position in hominoids, several have been suggested. The long clavicle found in hominoids can push the scapula to the dorsum of the trunk [1, 5, 6, 16]. The obliquely reorganized scapula found in hominoids can also potentially contribute to a dorsal scapula position since such a scapula can move closer to the dorsal midline [5, 17, 18].

It has also been suggested that the broad thorax of hominoids has contributed to their dorsal scapula since the thorax is an intrinsic part of the shoulder complex [1, 3–6, 16, 17, 19, 20]. However, the extent that a scapula can be pushed dorsally depends more on the length of the clavicle than on the cross-sectional shape of the thorax, since the length of the clavicle is the distance of the acromion of the scapula from the ventral midline. It is not entirely clear how the broad thorax contributes to the dorsal scapular position in hominoids, if it does at all. In humans with a hypoplastic clavicle, the scapula lies on the side of the broad thorax [21], instead of on the dorsum, which further attests to the fact that scapular position depends more on the clavicular length and less, if at all, on the cross-sectional shape of the thorax.

Chan [2] compared the scapular position of a wide range of primates with the vertebral end of the scapula resting on the third thoracic vertebra, the standardized shoulder configuration he used for the comparison. In that standardized shoulder configuration, the shoulder girdle can be visualized as a triangle (the so-called TSC triangle), with the three sides being the dorsoventral diameter of the thorax from the cranial end of the sternum to the posterior border of the third thoracic vertebral body (T in TSC), the length of the scapular from acromion to vertebral end of the scapular spine (S in TSC), and the clavicle from the cranial end of sternum to the acromioclavicular joint (C in TSC). The scapular position is therefore not totally determined by the length of the clavicle but is affected by the interactions of the three components of the shoulder girdle (the thorax, scapula, and clavicle). A longer clavicle will give rise to a more dorsal scapula and so will a shorter scapula and a shorter dorsoventral thoracic diameter, as defined above.

Chan's [2] analysis could potentially explain how a broad thorax can lead to a dorsal scapular position in hominoids: if the dorsoventral diameter of a broad thorax is shorter than that of a narrow thorax, then a more dorsally situated scapula can result. However, this explanation is true* if and only if* the area of the thoracic cross-section remains the same, because it is only then that a broad thorax will have a shorter dorsoventral diameter (area of an ellipse = *π* ∗ length of semimajor axis ∗ length of semiminor axis).

However, in Chan's study [2], it was found that the dorsoventral diameter of the hominoid thorax is actually* longer* than that in the arboreal quadrupedal monkeys, after scaling for the differences in the animals' size. There are three mutually exclusive hypotheses that can explain this observation.

- If the cranial thorax of hominoids has the same cross-sectional shape as that of arboreal quadrupedal monkeys, the longer dorsoventral thoracic diameter in hominoids can be explained by their larger cranial thorax with larger cross-sectional areas, leading to longer transverse and dorsoventral diameters.
- If the cranial thorax of hominoids is broader than that of arboreal quadrupedal monkeys, the hominoids' longer dorsoventral thoracic diameter can be explained by a much larger cranial thorax. Their cranial thorax needs to be even bigger than that postulated in the first hypothesis, in order for the increase in dorsoventral diameter due to an increase in the volume of the cranial thorax to compensate for the decrease in the dorsoventral diameter due to the broadening of the cross-sectional shape.
- If the cranial thorax of hominoids is less broad (narrower) compared to that of arboreal quadrupedal monkeys, the hominoids' longer dorsoventral thoracic diameter can simply be due to the difference in cross-sectional shape, even if there is no difference in volume of the cranial thorax.

Previously published data on thoracic shape did not allow a meaningful comparison of the thoracic shape of primates in order to differentiate the above three explanations. The few data available were based on measurements of the external surface of the body at the level of the sternal end of the fourth rib [6, 22–24]. These data indicate not the shape of the bony thorax but the shape of the torso, which is influenced by the thickness of pelt, subcutaneous tissue, muscle bulk, and the length of spinous processes. Moreover, these data document only the shape of the torso at a single thoracic level, neglecting the variation of thoracic shape at different thoracic levels in the same animal. Of particular interest is the upper thorax, which is functionally more important in determining arm mobility since the scapula glides on this part of the thorax [25]. The method used by Schultz [6, 22, 23] cannot ascertain the dimensions of the upper thorax because it is embedded in the musculature of the shoulder girdle.

In addition, previous studies have focused on the cross-sectional shape of the thorax alone, neglecting the regional variation of thoracic volume [3, 6, 22, 23, 26]. The dorsoventral thoracic diameter, which affects scapular position, depends on both the cross-sectional shape* and *the size of the thorax at the scapular level. Therefore, a functional analysis of thoracic morphology should include the two parameters.

*(a) Cross-Sectional Shape.* The cross-sectional shape of the primate thorax is expressed by the thoracic index, which is the ratio of transverse diameter to the dorsoventral diameter, multiplied by 100. A thoracic index above 100 implies that the transverse diameter is longer than the dorsoventral diameter (i.e., dorsoventrally compressed) and has been described as “broad” or “wide.” And a thoracic index below 100 indicates lateral compression and will be described as “narrow.” It must be noted that these terms of “broad,” “wide,” and “narrow” refer to the cross-sectional shape of a thoracic section and do not refer to the width, be it scaled or not scaled to the size of the animal.

*(b) Volume Distribution.* This refers to the relative volume of the cranial and caudal regions of the thorax. In a large cranial thorax, the area of a cross-section in that region will also be large. Terms for describing these features are those used in ordinary English language for volume: “large” and “small.” This is a feature that is independent of the cross-sectional shape. For example, in a thorax with a large cranial thorax, the cross-sectional shape of that region can be either broad or narrow.

Currently, no satisfactory data exist on regional cross-sectional shape and volume distribution. This study aims, for the first time in the study of the primate thorax, to collect such data in order to test the above three hypotheses for explaining the longer dorsoventral diameter of the cranial thorax of hominoids.

## 2. Materials and Methods

Table 1 lists all the species and the number of cadavers of each species included in the present study. Only cadavers with fully erupted teeth were used, since thoracic shape changes with the maturity of the animal [23]. Only cadavers with an unopened chest wall were used. Excluded from the study were cadavers with obvious deformities, for example, crushed thoraces, excessive flexion, or curvature of the thoracic vertebral column. All specimens were fluid preserved, except for the four Homo sapiens specimens, which were dried and mounted skeletons. It was assumed that deformation of the thorax during preparation of such large skeletons is insignificant.

A biplanar radiographic method was used to collect data on cross-sectional shape and relative volume distribution of the thorax on preserved cadavers.

### 2.1. Measurement of Thoracic Cross-Sectional Shape

For each of these cadavers, one anteroposterior and one lateral radiograph were taken. The X-ray machines used were a Picker portable industrial machine (in the Smithsonian Institution) and an Acoma Super-80 portable X-ray unit (in the American Museum of Natural History). Kodak diagnostic ready-pack X-ray film (XTL-2) was used. The X-ray source was positioned at least one meter from the specimen and the X-ray film was placed immediately behind the specimen.

**Figure 1:**
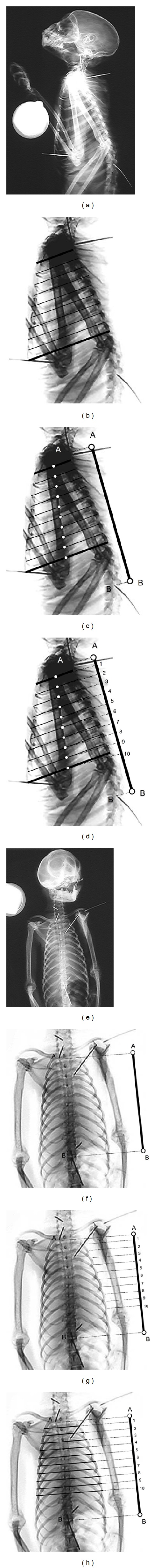
Radiographs illustrating the steps used in measuring the dorsoventral and transverse thoracic diameters for calculating thoracic indices at the decisternal levels. See text for the procedures involved in measuring the dorsoventral and transverse thoracic diameters.

For the lateral radiograph (Figure 1(a)), the cadaver was laid on its side and its head was propped up so that the mid-sagittal plane of the thorax (containing the sternum and the thoracic vertebral column) was parallel to the X-ray film. The thorax was centered on the film with the X-ray source perpendicular to the film directly above it. For the anteroposterior radiograph (Figure 1(e)), the cadaver was laid on its back so that the mid-coronal plane of the thorax was parallel to the X-ray film.

A radio-opaque scale was included in each radiograph for calculating the magnification factor, which in turn allows the calculation of the true thoracic diameter from the diameter measured on the radiograph. The radio-opaque scale was placed parallel to the X-ray film in the mid-sagittal plane (in the lateral radiograph) or in the mid-coronal plane of the thorax (in the anteroposterior radiograph).

The following method was used to measure the dorsoventral and transverse diameters, respectively, at 10 levels on the lateral and anteroposterior radiographs.

1. On the lateral radiograph (Figure 1(b)), a line was extended posteriorly from the caudal end of the sternum perpendicular to the spine (or the tangent of the spine, if it is curved at the lower thoracic region). Another line was extended posteriorly from the cranial end of the sternum, parallel to the first line. Eight equally spaced lines were drawn between the first two lines. The resulting 10 parallel lines represent the 10 thoracic levels (hereafter called decisternal levels) at which the dorsoventral thoracic diameters were measured, between the sternum and the dorsal border of the rib cage (Figure 1(b)).
2. The midpoints of these 10 dorsoventral thoracic diameters were projected perpendicularly onto a reference line (AB in Figure 1(c), where A and B were the tips of metal pins inserted into the back of the cadaver) and their positions measured from point A (Figure 1(d)).
3. On the anteroposterior radiograph of the same cadaver (Figure 1(f)), the positions of the 10 thoracic levels were marked on the reference line AB (Figure 1(g)) (after taking into consideration the magnification factor, which can be calculated from the lengths of AB in the lateral and the anteroposterior radiographs). Ten horizontal lines were drawn at these 10 levels (Figure 1(h)), at which the 10 transverse diameters were measured, corresponding, respectively, to the 10 dorsoventral diameters measured on the anteroposterior radiographs.

For each of the decisternal levels, the thoracic index was calculated from the transverse and dorsoventral diameter thus obtained. The above method has been tested on a Styrofoam asymmetrical cone-shaped model of the thorax. The true thoracic indices can be calculated from direct measurements on the model. It was found that the thoracic indices calculated using the above method had a mean percent error of 3.3% (error from the true indices) and a mean percent deviation of 2.8% (deviation of multiple measurements from the sample mean index).

One should note that only the part of the thorax posterior to the sternum was included in this study. The scapula usually moves on the cranial half of this part of the thorax [25]. One should note that the part of the thorax caudal to the tenth decisternal level was not included since it lacks an anterior border, it is probably functionally less important, and its shape is too easily affected by the process of preservation.

### 2.2. Measurement of Thoracic Volume Distribution

The volume distribution of the thorax was found by calculating the relative volume of the nine thoracic* segments* bounded by the 10 equally spaced decisternal* levels*. The first thoracic segment has the first and second decisternal levels as its upper and bottom planes, respectively, while the second segment has the second and third levels, and so on. In calculating their volume, each segment was approximated as a column with a constant oval cross-sectional area, the major and minor axes of which were the averaged transverse and dorsoventral thoracic diameters of the cross-sections bounding that thoracic segment (the transverse and dorsoventral diameters are interchangeable in the calculation and therefore it does not matter which diameter is the major axis and which is the minor axis):

(1) Volume  of  each  thoracic  segment =area  of  the  oval  cross-section∗height =(π∗a∗b)∗h a=semimajor  axis=transverse  diameter2   b=semiminor  axis=dorsoventral  diameter2 h=height  of  each  segment.

The volume of each segment was then expressed as a percentage of the total volume of all the nine segments. Since only relative volume was calculated and all the thoracic segments of a particular specimen have the same height (the decisternal levels are equally spaced), the height of each segment did not need to be measured since it was eliminated during the calculation. The segmental relative volumes were then plotted against the segment level to obtain a volume distribution curve, the slope of which was found by simple linear regression (least-squares curve fitting).

Mann-Whitney tests were used in the following comparisons that involved two samples and Kruskal-Wallis tests for those that involved more than two samples. These nonparametric statistical methods were used since they do not assume normal distributions of data points [27, 28], which cannot be tested for the small samples collected in this study. These nonparametric tests, however, are less powerful than tests that assume normal distribution of data [28].

## 3. Results

### 3.1. Cross-Sectional Shape

**Figure 2:**
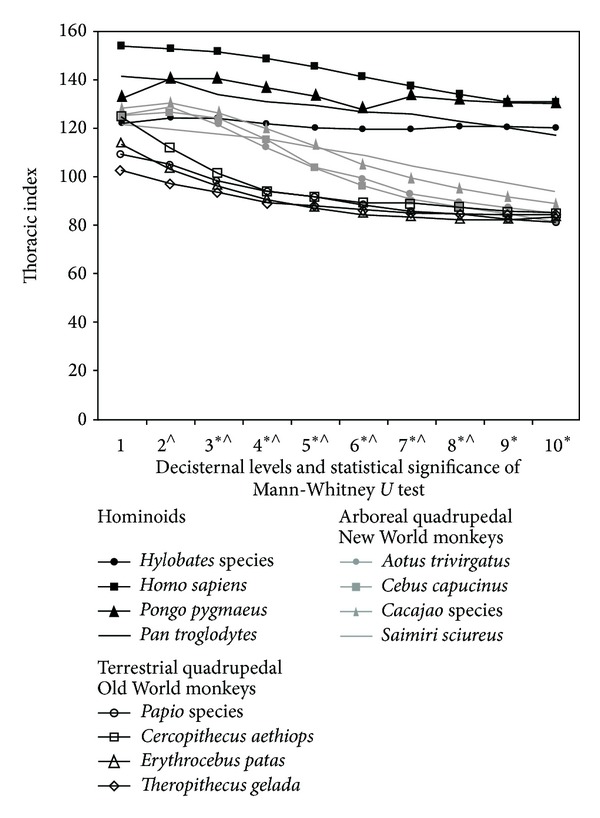
Means of the thoracic indices at all decisternal levels of the four hominoids, the four arboreal quadrupedal New World monkeys, and the four terrestrial quadrupedal Old World monkeys included in this study. Also shown were the results of Mann-Whitney tests comparing the species means (∗ : 0.05 ≥ *P* ≥ 0.01 for the comparison between hominoids and the arboreal quadrupedal New World monkeys; ∧:0.05 ≥ *P* ≥ 0.01 for the comparison between the arboreal quadrupedal New World monkeys and the terrestrial quadrupedal Old World monkeys).

Figure 2 shows the thoracic indices of hominoids, arboreal quadrupedal New World monkeys, and terrestrial quadrupedal Old World monkeys. In hominoids, the thoracic index remains above 120 at all decisternal levels, meaning that both the cranial and the caudal parts of the thorax are broad. In arboreal quadrupedal New World monkeys, the cranial thorax is also broad, with the thoracic index between 120 and 127, while the caudal thorax is narrow, with thoracic index between 81 and 93. Except for the first 2 decisternal levels, hominoids have higher thoracic indices than arboreal quadrupedal New World monkeys (Figure 2).

In terrestrial quadrupedal Old World monkeys, the thoracic index starts at above 100 (between 101 to 123) at the first decisternal level and then decreases from the cranial to the caudal region of the thorax, reaching values between 80 and 90 at about the sixth decisternal level and then remaining more or less constant (Figure 2). The arboreal quadrupedal New World monkeys have significantly higher thoracic indices from the second decisternal level to the eighth decisternal level. In the two most caudal levels, the thoracic indices of the two groups are very similar.

### 3.2. Volume Distribution

**Figure 3:**
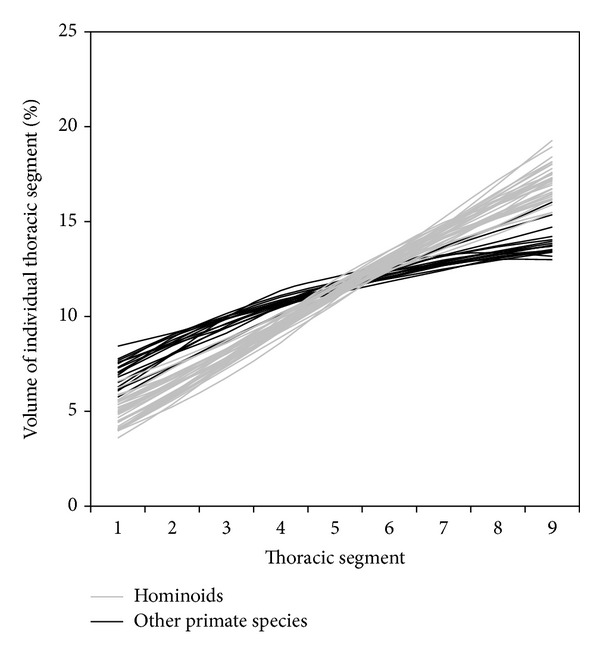
The thoracic volume distribution curves for all specimens. The vertical axis is the relative volume of each thoracic segment between two adjacent decisternal levels. It has no units.

**Figure 4:**
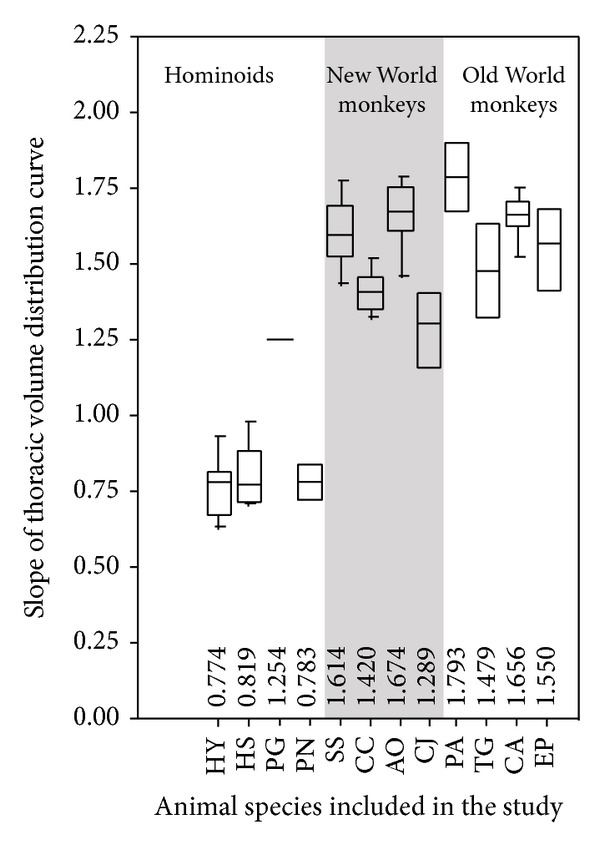
Quartile box plot of the slopes of the thoracic volume distribution curves for all species included in the study. See Table 1 for species abbreviations.

Figure 3 shows the volume distribution curve of all the individual specimens in the present study to show the variation in their volume distribution.  Figure 4 is a quartile box plot of the slope of the thoracic volume distribution curves for all included species.

It is noticed that the volume distribution curves intersect approximately at the fifth thoracic segment. This is simply a geometric consequence. The* y*-values (the percentage volumes) of all the points on a curve must add up to a total of 100%; that is, the area under all curves must be the same and equal to 100%. And these curves are all approximately straight lines, because percentage volume changes gradually from the cranial segments to the caudal segments, although the rate of change may differ among different species. Therefore, for any two curves, if one is higher than the other in the cranial region, it must be lower than the other curve in the caudal region for both of them to have the same area (100%) under them. The two curves must therefore intersect at approximately the fifth thoracic segment (not “exactly” at the fifth thoracic segment because these curves are only “approximately” straight lines).

The slope of the thoracic volume distribution curve varies significantly among primate species (Kruskal-Wallis test *P* < 0.0001) (Figure 4). Hominoids were found to have the lowest slopes in their volume distribution curve (Figures 3 and 4); that is, in the cranial decisternal levels, the hominoid curves lie above the curves of other primates. In other words, the relative volume of the cranial thorax of hominoids is the highest among primates. The slope did not differ significantly among the four included hominoid species (*P* = 0.40), although it is observed that the thorax of humans and gibbons tends to be barrel shaped while that of the other apes tends to be cone shaped (but note that these terms of barrel-shaped and cone-shaped thorax are based on observation from the frontal plane only and of the whole thorax, not just the sternal part of the thorax). There was no significant difference in slope between the arboreal quadrupedal New World monkeys and the terrestrial quadrupedal Old World monkeys (*P* = 0.49).

## 4. Discussion

### 4.1. Locomotor Adaptations of the Hominoid Thorax

#### 4.1.1. The Cranial Thorax

This study has shown that the cranial thorax of hominoids is larger and broader (except in the first two decisternal levels) than that of other primates. These results support the second hypothesis postulated in the Introduction for explaining the longer dorsoventral diameter in the cranial thorax of hominoids. An increase in volume without changes in cross-sectional shape causes an increase in the dorsoventral diameter, while broadening of the thorax without changes in the volume causes a decrease in dorsoventral diameter. In hominoids, the increase in dorsoventral diameter caused by the increase in the volume of the cranial thorax has overcompensated for the decrease caused by the broadening of the cranial thorax. This has led to a net increase in the dorsoventral diameter of the cranial thorax in hominoids, as found in Chan's study [2].

The longer dorsoventral diameter of the cranial thorax in hominoids did not contribute to the dorsal scapular position [2]. Although the larger and broader cranial thorax of hominoids does not contribute to their dorsal scapula, it could still have an adaptive role in their locomotion. In the study by Jenkins Jr. et al. [25], it was shown that when spider monkeys brachiate, the scapula glides on the dorsal surface of the cranial thorax. The large and broad cranial thorax in hominoids can provide a large surface in the coronal plane for the gliding of the scapula.

#### 4.1.2. The Caudal Thorax

Hunt [17] suggested that the hominoid thorax is an adaptation for suspensory behavior. He argued that a broad thorax can reduce the compressive force on the bony thorax because the more coronal orientation of the muscles connecting the arm and the trunk reduces the compressive component on the thorax. But another important consequence is that the muscle tension needed to suspend the animal is reduced. A broad thorax will bring the muscles into a more coronal orientation and therefore more in line to counter the body weight. Therefore, muscle tension does not need to be as great in order to generate a vertical component equal to the body weight. Reduced energy expenditure in suspensory behavior can be an adaptive advantage.

Data collected in the current study support Hunt's [17] hypothesis. The caudal region of the hominoid thorax has a lower relative volume and is broad compared to that of arboreal quadrupedal New World monkeys and the terrestrial quadrupedal Old World monkeys, meaning that the dorsoventral diameters in the caudal thorax are shorter in hominoids. The shorter dorsoventral thoracic diameters can reduce the energy expenditure in suspensory behavior for the reasons stated above.

However, one should always be cautious in advancing an adaptive explanation of morphology [29]. The broad caudal thorax in hominoids may not have a functional adaptive value at all. In hominoids, the lumbar spine is short [30, 31] and part of the latissimus dorsi arises from the iliac crest [24]. Such features may protect the lumbar spine from excessive lateral bending during latissimus dorsi contraction. But the close proximity of the caudal thorax to the pelvis may necessitate changes in the shape of the caudal thorax in response to changes in the pelvis. Straus and Wislocki [26] even suggested that a broad thorax can be caused by an orthograde posture simply due to gravity.

### 4.2. Respiratory Adaptation of the Hominoid Thorax

The thorax contains the lungs, and changes in the shape of the thorax can potentially bring along changes in the distribution of lung tissue within the thorax. The greater volume of the cranial thorax in hominoids may imply that more lung tissue is present in the cranial region and therefore the respiratory function may be different.

In humans, both ventilation and perfusion increase from the cranial to the caudal region of the orthograde lung. For ventilation, it is because the pleural pressure is higher (less negative) surrounding the lower part of the lung [32, 33]. Due to the characteristic elastic behavior of pulmonary tissue, a given decrease in pleural pressure during inspiration will cause more expansion in the pulmonary tissue in the caudal part of the lung than in the cranial region. Pulmonary perfusion also increases from the cranial to the caudal regions of an orthograde lung because of the vertical hydrostatic pressure gradient in the pulmonary blood vessels due to the effect of gravity. This pressure gradient is quite significant for the pulmonary circulatory system, which is a relatively low pressure system. Although both ventilation and perfusion increase from the cranial to the basal region, perfusion increases faster than ventilation, with the result that the ventilation-perfusion ratio* decreases* in going from the cranial to the caudal region [33].

The ventilation-perfusion ratio determines gaseous exchanges [33]. For a pulmonary unit with a zero ventilation-perfusion ratio, that is, perfusion without ventilation, the partial pressure of oxygen and carbon dioxide in the end-capillary blood will be the same as that of the incoming mixed venous blood, that is, no gaseous exchange. As the ventilation-perfusion ratio increases, the end-capillary oxygen partial pressure increases while that of carbon dioxide decreases, thus approaching the pressure of these gases in the inspired air.

In the cranial region of an orthograde lung, the ventilation-perfusion ratio is high and the oxygen pressure in the end-capillary blood is high. In the caudal region, the ventilation-perfusion ratio is low and the oxygen pressure in the end-capillary blood is low. The mixing of blood from the cranial and the caudal regions results in a depression of the arterial oxygen partial pressure below that of the alveolar oxygen partial pressure. The difference between the arterial oxygen partial pressure and the alveolar oxygen partial pressure is called the “alveolar-arterial oxygen difference.”

The larger cranial thorax in hominoids means that more pulmonary tissue is present in the cranial region. Thus more oxygen-rich blood comes from the larger cranial region and less oxygen-poor blood comes from the smaller caudal region. The overall effect is a higher arterial oxygen partial pressure. If hominoids were to possess lungs shaped like those of pronograde primates, that is, with a lower volume of the cranial thorax, the alveolar-arterial oxygen difference would be greater. However, this hypothesis on the respiratory effects of cranial expansion in the hominoid thorax is based on human data, and whether extrapolation to other primates is valid remains to be tested. One would also expect to see similar thoracic adaptations in other primates and nonprimate mammals which spend significant time in orthograde posture. But more detailed data on their thoraces need to be available to test for such convergence.

## 5. Conclusions

In conclusion, this study has shown that the cranial thorax of hominoids is larger and broader (except in the first two decisternal levels) than that of other primates. These findings explain the longer dorsoventral diameter found in the cranial thorax of hominoids [2]: the increase in dorsoventral diameter caused by the increase in the volume of the cranial thorax overcompensates for the decrease caused by the broadening of the cranial thorax.

The large and broad cranial thorax of hominoids does not contribute to the dorsal scapular position in hominoid since they result in a longer dorsoventral diameter but can serve as a dorsal gliding surface for the scapula. The large cranial thorax can also be explained as a respiratory adaptation for orthograde posture in hominoids.

## Figures and Tables

**Table 1:** The species and the number of specimens for each species included in the present study.

Species	Abbreviations	Source^1^	Number of specimens
*Hylobates *species	HY	AMNH, SI, YK	8
*Homo sapiens *	HS	DU	6
*Pongo pygmaeus *	PG	YK	1
*Pan troglodytes *	PN	BM	2
*Saimiri sciureus *	SS	SI	9
*Cebus capucinus *	CC	SI	5
*Aotus trivirgatus *	AO	SI	5
*Cacajao rubicundus *	CJ	SI	3
* Papio anubis *	PA	SI, AMNH	2
*Theropithecus gelada *	TG	SI	4
*Cercopithecus aethiops *	CA	SI	6
*Erythrocebus patas *	EP	SI	4

^1^AMNH: American Museum of Natural History; BM: Buckshire Co. and Milwaukee Zoo; DU: Duke University, Department of Biological Anthropology and Anatomy; SI: United States National Museum of Natural History, Smithsonian Institution; YK: Yerkes Primate Center.
